# Supplementary material for: Resolving the full spectrum of human genome variation using Linked-Reads
Source: Genome Res. 2019 Apr;29(4):635–45. doi: 10.1101/gr.234443.118 (PMC6442396; doi:10.1101/gr.234443.118)
Supplement: Supplemental Material [file supp_gr.234443.118_Supplemental_longranger-2.2.zip › longranger-2.2.tar.bz2!/lr2.2-public/lib/stan/cmdstan/stan/doc/api/html/classstan_1_1prob_1_1welford__covar__estimator.html]

Stan: stan::prob::welford\_covar\_estimator Class Reference


|  |  |
| --- | --- |
|  | Stan  2.5.0  probability, sampling & optimization |


- Main Page
- Related Pages
- Namespaces
- Classes
- Files

- Class List
- Class Index
- Class Hierarchy
- Class Members

All Classes Namespaces Files Functions Variables Typedefs Enumerations Enumerator Friends Macros Pages

- stan
- prob
- welford\_covar\_estimator

Public Member Functions |
Protected Attributes |
List of all members

stan::prob::welford\_covar\_estimator Class Reference

`#include <welford_covar_estimator.hpp>`

|  |  |
| --- | --- |
| Public Member Functions | |
|  | welford\_covar\_estimator (int n) |
|  | |
| void | restart () |
|  | |
| void | add\_sample (const Eigen::VectorXd &q) |
|  | |
| int | num\_samples () |
|  | |
| void | sample\_mean (Eigen::VectorXd &mean) |
|  | |
| void | sample\_covariance (Eigen::MatrixXd &covar) |
|  | |

|  |  |
| --- | --- |
| Protected Attributes | |
| double | \_num\_samples |
|  | |
| Eigen::VectorXd | \_m |
|  | |
| Eigen::MatrixXd | \_m2 |
|  | |

## Detailed Description

Definition at line 11 of file welford\_covar\_estimator.hpp.

## Constructor & Destructor Documentation

|  |  |  |  |  |  |
| --- | --- | --- | --- | --- | --- |
| stan::prob::welford\_covar\_estimator::welford\_covar\_estimator | ( | int | *n* | ) |  |

inline

Definition at line 15 of file welford\_covar\_estimator.hpp.

## Member Function Documentation

|  |  |  |  |  |  |
| --- | --- | --- | --- | --- | --- |
| void stan::prob::welford\_covar\_estimator::add\_sample | ( | const Eigen::VectorXd & | *q* | ) |  |

inline

Definition at line 25 of file welford\_covar\_estimator.hpp.

|  |  |  |  |  |
| --- | --- | --- | --- | --- |
| int stan::prob::welford\_covar\_estimator::num\_samples | ( |  | ) |  |

inline

Definition at line 35 of file welford\_covar\_estimator.hpp.

|  |  |  |  |  |
| --- | --- | --- | --- | --- |
| void stan::prob::welford\_covar\_estimator::restart | ( |  | ) |  |

inline

Definition at line 19 of file welford\_covar\_estimator.hpp.

|  |  |  |  |  |  |
| --- | --- | --- | --- | --- | --- |
| void stan::prob::welford\_covar\_estimator::sample\_covariance | ( | Eigen::MatrixXd & | *covar* | ) |  |

inline

Definition at line 39 of file welford\_covar\_estimator.hpp.

|  |  |  |  |  |  |
| --- | --- | --- | --- | --- | --- |
| void stan::prob::welford\_covar\_estimator::sample\_mean | ( | Eigen::VectorXd & | *mean* | ) |  |

inline

Definition at line 37 of file welford\_covar\_estimator.hpp.

## Member Data Documentation

Eigen::VectorXd stan::prob::welford\_covar\_estimator::\_m

protected

Definition at line 48 of file welford\_covar\_estimator.hpp.

Eigen::MatrixXd stan::prob::welford\_covar\_estimator::\_m2

protected

Definition at line 49 of file welford\_covar\_estimator.hpp.

double stan::prob::welford\_covar\_estimator::\_num\_samples

protected

Definition at line 46 of file welford\_covar\_estimator.hpp.

---

The documentation for this class was generated from the following file:

- src/stan/prob/welford\_covar\_estimator.hpp


---

[ Stan Home Page ]

*© 2011–2014,
Stan Development Team.*
